# The Association between Four Genetic Variants in MicroRNAs (rs11614913, rs2910164, rs3746444, rs2292832) and Cancer Risk: Evidence from Published Studies

**DOI:** 10.1371/journal.pone.0049032

**Published:** 2012-11-14

**Authors:** Bangshun He, Yuqin Pan, William C. Cho, Yeqiong Xu, Ling Gu, Zhenglin Nie, Liping Chen, Guoqi Song, Tianyi Gao, Rui Li, Shukui Wang

**Affiliations:** 1 Central Laboratory of Nanjing First Hospital, Nanjing Medical University, Nanjing, China; 2 Department of Clinical Oncology, Queen Elizabeth Hospital, Kowloon, Hong Kong; University of Aberdeen, United Kingdom

## Abstract

MicroRNAs (miRNAs) participate in diverse biological pathways and may act as either tumor suppressor genes or oncogenes. Single nucleotide polymorphisms (SNPs) in miRNA may contribute to cancer development with changes in the microRNA's properties and/or maturation. Polymorphisms in miRNAs have been suggested in predisposition to cancer risk; however, accumulated studies have shown inconsistent conslusionss. To further validate determine whether there is any potential association between the four common SNPs (miR-196a2C>T, rs11614913; miR-146aG>C, rs2910164; miR-499A>G, rs3746444; miR-149C>T, rs2292832) and the risk for developing risk, a meta-analysis was performed according to the 40 published case-control studies. Odds ratios (ORs) with 95% confidence intervals (CIs) were calculated to assess the extent of the association. The results demonstrated that the rs11614913TT genotype was significantly associated with a decreased cancer risk, in particular with a decreased risk for colorectal cancer and lung cancer, or for Asian population subgroup. In addition, the rs2910164C allele was associated with decreased risk for esophageal cancer, cervical cancer, prostate cancer, and hepatocellular carcinoma (HCC), in particular in Asian population subgroup. Similarly, the rs3746444G allele was observed as a risk factor for cancers in the Asian population. It is concluded that two SNPs prsent in miRNAs(rs11614913TT, and rs2910164C) may protect against the pathogenesis of some cancers, and that the rs3746444 may increase risk for cancer.

## Introduction

MicroRNAs (miRNAs) are small, single-stranded, 19–21 nucleotide long non-protein-coding RNA molecules, functioning as negative regulators that involve post-transcriptional gene expression through binding to their target mRNAs regions and consequently lead to mRNA cleavage or translational repression [1]. Accumulating evidence has shown that miRNAs regulate the expression of roughly 10–30% of the all human genes through post-transcriptional mechanisms [2], contributing to excessive physiologic and pathologic conditions, including cell differentiation, proliferation, and apoptosis [1], and inparticular to the development and progression of various human cancers by regulating the expression of proto-oncogenes or tumor suppressor genes [3], [4], [5].

SNPs in miRNA genes are regarded to affect function by three ways: first, through the transcription of the primary transcript; second, through pri-miRNA and pre-miRNA processing; and third, through effects on miRNA-mRNA interactions [6]. Recently, several studies have demonstrated that some polymorphism(SNPs) present in the miRNA genes, which can alter miRNA expression and/or maturation and be associated with the development and progression of cancer [6]. For example, four SNPs – miR-196a2C>T (or rs11614913), miR-146aG>C (rs2910164), miR-499A>G (rs3746444), and miR-149C>T (rs2292832) – identified in the pre-miRNA regions of miR-146a, miR-149, miR-196a2, and miR-499, respectively, have been reported to be associated with cancer risk [7], [8]. However, conclusions of the relevant studies remain inconsistent, in part because of heterogeneity of the cancer subtype, small sample size, and ethnicity of the patients. To further determine whether there is an association of the four SNPs in the miRNA genes with the risk for developing cancer, a comprehensive review and analysis of published data from different studies is needed. In this study, we have extensively reviewed literature and performed a meta-analysis based on all eligible case-control published data to evaluate the association between the four polymorphisms and cancer susceptibility.

## Materials and Methods

### Identification of eligible studies

We carried out a search of the PubMed and Embase databases for all relevant reports using the key words ‘microRNA/miR-146a/miR-149/miR-196a2/miR-499’, ‘polymorphism’, and ‘cancer’ (updated to Jun 23, 2012). The search was limited to English language papers and human subject studies. We evaluated potentially relevant publications by examining their titles and abstracts, thereafter all studies matching the eligible inclusion criteria were retrieved. In addition, studies were identified by a manual search of the references listed in the reviews involved. All the studies were included if they met the following criteria: (i) about the rs11614913, rs2910164, rs3746444, and rs2292832 polymorphisms and cancer risk, (ii) from a case–control designed study, and (iii) genotype frequencies available.

### Data extraction

All data complying with the selection criteria were extracted independently by two staff (B.S.H., and Y.Q.X). For each study, the following characteristics were extracted: the first author's last name, year of publication, country of origin, ethnicity, the numbers of genotyped cases and controls, source of control groups (population- or hospital-based controls), genotyping methods and cancer type. Ethnic descents were categorized as Caucasian, Asian or mixed (which included more than one ethnic descent). One study included the information for genotype rs11614913 CT+TT, without the data for CT and TT genotypes, so we were only able to calculate the OR for the comparison between CT+TT vs. TT [9].

### Statistical analysis

The four SNPs in miRNAs were tested for the associations with cancer susceptibility based on different genetic models. The meta-analysis examined the overall association of the four SNPs with the risk of cancer as measured by odds ratios (ORs) at the 95% confidence intervals (CIs). To contrast the wild-type homozygote (WW), we first estimated the risk of the rare allele homozygote (RR) and heterozygous (WR) genotypes on cancers, then evaluated the risk of cancer under a dominant model (RR+WR vs. WW). In addition, recessive model associations were also estimated (RR vs. WR+WW). Moreover, stratified analyses were also performed by ethnicity (Asian, and Caucasian), cancer type (if only one cancer type contained fewer than two individual studies it was combined into the ‘Other Cancers’ group) and source of control for rs11614913 and rs2910164. Stratified analyses were performed by ethnicity for rs2292382, and by ethnicity and cancer type for rs3746444, respectively.

The statistical significance of the pooled OR was determined with the Z test, and a P value of <0.05 was considered significant. The heterogeneity between studies was evaluated by the Chi-square based Q statistical test [10], with heterogeneity (*P*_h_) <0.05 being considered significant. A fixed-effect model using the Mantel–Haenszel method and a random-effects model using the DerSimonian and Laird method were used to pool the data [11]. The random-effects model was used when heterogeneity in the results of the studies was found; otherwise the fixed-effect model was used. Sensitivity analyses were performed to assess the stability of the results, namely, a single study in the meta-analysis was deleted each time to reflect the influence of the an individual data set on the pooled OR. To determine whether there was a publication bias, Funnel plots and Egger's linear regression tests were applied [12].

All statistical tests for this meta-analysis were performed with STATA version 10.0 (Stata Corporation College Station, TX, USA).

## Results

### Characteristics of the studies

A total of 40 eligible studies met the prespecified inclusion criteria (See Figure S1), in which 27, 26, 13, and 6 studies were pooleded for the analyses of the rs11614913, rs2910164, rs37464444, and rs2292832, respectively (Table 1). All studies were case-control studies, including 8 studies on hepatocellular cancer (HCC), 5 breast cancer, 5 gastric cancer, 4 colorectal cancer, 3 lung cancer, and 15 on other cancer types, and one on breast/ovarian cancer was enrolled. There were 28 studies of Asian descendent, 11 of Caucasian descendents and one of mixed ethnicity [13]. To determine the SNPs, genotyping by polymerase chain reaction-restriction fragment length polymorphism (PCR-RFLP) and TaqMan assay were performed in the 28 studies. In addition, 34 studies were included based on the control sex- and age-matched for the case groups (six studies with 2,050 cases and 2,626 controls were not matched by age or sex), of which 33 were population-based and seven were hospital-based.

**Table 1:** Summary of published studies included.

	Author	Year	Race	Cancer type	Control	Method	Case/control	Polymorphism site
1	Xu	2008	Asian	HCC	PB	PCR-RFLP	479/504	rs2910164
2	Hu	2008	Asian	Breast Cancer	PB	PCR-RFLP	1009/1093	rs11614913,rs2910164,rs3746444,rs2292832
3	Jazdzewski	2008	Caucasian	Papillary thyroid carcinoma	PB	SNPshot	608/901	rs2910164
4	Ye	2008	Caucasian	Esophageal Cancer	PB	SNPlex assay	307/388	rs11614913,rs2910164
5	Horikawa	2008	Caucasian	Renal cell carcinoma	PB	SNPlex assay	276/277	rs11614913,rs2910164
6	Tian	2009	Asian	Lung Cancer	PB	PCR-RFLP	1058/1035	rs11614913,rs2910164,rs3746444,rs2292832
7	Hoffman	2009	mix	Breast Cancer	HB	iPLEX GOLD	426/466	rs11614913
8	Xu	2010	Asian	Prostate Cancer	PB	PCR-RFLP	251/280	rs2910164
9	Yoo	2010	Asian	lung cancer	PB	melting-curve analysis	654/640	rs11614913
10	Guo	2010	Asian	Esophageal cancer	PB	SNPshot	444/468	rs2910164
11	Dou	2010	Asian	Glioma	PB	LDR	643/656	rs11614913
12	Li	2010	Asian	HCC	HB	PCR-RFLP	310/222	rs11614913
13	Chen	2010	Asian	CRC	PB	LDR	126/407	rs11614913
14	Pastrello	2010	Caucasian	Breast/ovarian cancer	PB	PCR-RFLP	101/155	rs2910164
15	Qi	2010	Asian	HCC	PB	LDR	361/391	rs11614913
16	Peng	2010	Asian	Gastric Cancer	PB	PCR-RFLP	213/213	rs11614913
17	Srivastava	2010	Asian	Gallbladder cancer	PB	PCR-RFLP	230/230	rs11614913,rs2910164,rs3746444
18	Zeng	2010	Asian	Gastric Cancer	HB	PCR-RFLP	304/304	rs2910164
19	Catucci	2010	Caucasian	Breast Cancer	PB	Taqman	1852/2739	rs11614913,rs2910164,rs3746444
20	Liu	2010	Caucasian	Head and neck cancer	PB	PCR-RFLP	1109/1130	rs11614913,rs2910164,rs3746444,rs2292832
21	Christensen	2010	Caucasian	Head and neck cancer	PB	Taqman	484/555	rs11614913
22	Okubo	2011	Asian	Gastric Cancer	HB	PCR-RFLP	552/697	rs11614913,rs2910164,rs3746444
23	Zhou	2011	Asian	Cervical cancer	PB	PCR-RFLP	226/309	rs11614913,rs2910164,rs3746444
24	Akkız	2011	Caucasian	HCC	PB	PCR-RFLP	185/185	rs11614913
25	Zhu	2011	Asian	CRC	PB	Taqman	573/588	rs11614913
26	Permuth-Wey	2011	Caucasian	Glioma	PB	Illumina's Golden Gate	593/614	rs2910164
27	Zhan	2011	Asian	CRC	HB	PCR-RFLP	252/543	rs11614913
28	Hong	2011	Asian	Lung Cancer	PB	Taqman	406/428	rs11614913
29	Zhou	2011	Asian	Primary Liver Cancer	PB	PCR-RFLP		rs2910164,rs3746444
30	Min	2011	Asian	CRC	PB	PCR-RFLP	446/502	rs11614913,rs2910164,rs3746444,rs2292832
31	Hishida	2011	Asian	Gastric Cancer	HB	PCR-CTPP	583/1637	rs2910164
32	George	2011	Asian	Prostate cancer	PB	PCR-RFLP	159/230	rs11614913,rs2910164,rs3746444
33	Mittal	2011	Asian	Bladder Cancer	PB	PCR-RFLP	212/250	rs11614913,rs2910164,rs3746444
34	Akkız	2011	Caucasian	HCC	PB	PCR-RFLP	222/222	rs2910164
35	Yue	2011	Asian	Cervical cancer	PB	PCR-RFLP	447/443	rs2910164
36	Zhang	2011	Asian	Breast Cancer	PB	PCR-RFLP	248/243	rs11614913,rs2292832
37	Jedlinski	2011	Caucasian	Breast Cancer	PB	PCR-RFLP	187/171	rs11614913
38	Zhou	2012	Asian	Gastric Cancer	HB	Taqman	1686/1895	rs2910164
39	Xiang	2012	Asian	HCC	PB	PCR-RFLP	100/90	rs2910164,rs3746444
40	Kim	2012	Asian	HCC	PB	PCR-RFLP	159/201	rs11614913,rs2910164,rs3746444,rs2292832

HB, hospital based; PB, population based; HCC, hepatocellular carcinoma; CRC, colorectal cancer; PCR-RFLP, polymerase chain reaction–restriction fragment length polymorphism; PCR-CTPP, polymerase chain reaction with confronting two-pair primers; LDR, ligation detection reaction.

### Quantitative synthesis

For rs11614913 polymorphism, significant differences were observed for the comparison of TT vs. CC and TT vs. CC+CT. When grouped by the cancer types, significant associations were still found in colorectal cancer (TT vs. CC: OR = 0.70, 95% CI: 0.57–0.85, *P*_h_ = 0.284; TT+TC vs. CC: OR = 0.77, 95% CI: 0.65–0.91, *P*_h_ = 0.377; TT vs. CC+TC: OR = 0.80, 95% CI: 0.69–0.94, *P*_h_ = 0.198), lung cancer(TT vs. CC: OR = 0.77, 95% CI: 0.65–0.91, *P*_h_ = 0.284; TT+TC vs. CC: OR = 0.85, 95% CI: 0.74–0.98, *P*_h_ = 0.289; TT vs. CC+TC: OR = 0.83, 95% CI: 0.73–0.95, *P*_h_ = 0.281). In addition to the decreased risk for colorectal cancer and lung cancer, a decreased risk was also observed in other cancer groups (CT vs. CC: OR = 1.23, 95% CI: 1.10–2.13, *P*_h_ = 0.239; TT+CT vs. CC: OR = 1.13, 95% CI: 1.03–1.25, *P*_h_ = 0.096). Subgroup analysis by the ethnicity revealed a significant association in the comparison of TT vs. CC (OR = 0.80, 95% CI: 0.73–0.88, *P*_h_ = 0.169), and TT vs. CC+CT (OR = 0.85, 95% CI: 0.80–0.92, *P*_h_ = 0.300) in the Asian population. Subgroup analysis determined by the source of control revealed a significant association between the polymorphism and cancer risk in both the hospital and population based controls for the comparison of TT vs. CC and TT vs. CT+CC; moreover, a decreased risk was also observed for the comparison of TT+CT vs. CC in hospital based study, as summarized in Table 2.

**Table 2:** Stratification analyses of genetic susceptibility of rs11614913 polymorphism to cancer risk.

Category	Cases/Controls	TT vs. CC			CT vs. CC			TT+CT vs. CC			TT vs. CC+CT		
		OR(95% CI)	*P* a	*I* ^2^	OR(95% CI)	*P* a	*I* ^2^	OR (95% CI)	*P* a	*I* ^2^	OR(95% CI)	*P* a	*I* ^2^
Total	12663/14739	**0.83(0.74,0.93)** b	0.001	52.5	0.98(0.90,1.07)^b^	0.004	47.5	0.94(0.86,1.02)^b^	0.001	53.8	**0.86(0.79,0.95)** b	0.005	46.7
Cancer types													
Breast cancer	3722/4712	0.81(0.61,1.09)^b^	0.014	68	0.94(0.85,1.04)	0.532	0	**0.91(0.83,1.00)**	0.148	41	0.87(0.70,1.08)^b^	0.027	63.5
Colorectal cancer	1397/2040	**0.70(0.57,0.85)**	0.284	21.1	0.81(0.65,1.08)	0.367	5.2	**0.77(0.65,0.91)**	0.377	3.1	**0.80(0.69,0.94)**	0.198	35.7
HCC	1015/999	0.74(0.47,1.19)^b^	0.022	69	0.90(0.72,1.11)	0.631	0	0.85(0.69,1.04)	0.19	37	0.18(0.57,1.15)^b^	0.037	64.6
Lung cancer	2118/2103	**0.77(0.65,0.91)**	0.895	0	0.90(0.77,1.04)	0.098	57	**0.85(0.74,0.98)**	0.289	19.4	**0.83(0.73,0.95)**	0.281	21.3
Gastric cancer	765/910	0.80(0.61,1.06)	0.306	4.5	0.84(0.65,1.08)	0.163	48.5	0.82(0.65,1.04)	0.162	48.8	0.89(0.72,1.11)	0.698	0
Other cancers	3646/3975	1.06 (0.91,1.23)	0.125	38.2	**1.23(1.10,1.37)**	**0.239**	23.9	**1.13(1.03,1.25)**	0.096	40.7	0.93(0.74,1.17)	0.024	56.7
Ethnicities													
Asian	7837/8878	**0.80(0.73,0.88)**	0.169	23.7	0.99(0.88,1.13)^b^	0.001	57.4	0.95(0.84,1.07)^b^	0.001	58.3	**0.85(0.80,0.92)**	0.3	12.6
Caucasian	4400/5395	0.94(0.71,1.23)^b^	0.006	69.7	1.01(0.92,1.04)^b^	0.597	0	0.98(0.90,1.07)	0.181	32.3	0.94(0.74,1.21)^b^	0.005	70.5
Source of controls													
Population based	11123/12811	**0.87(0.77,0.98)** b	0.009	46.7	1.01(0.91,1.11)^b^	0.002	52.4	0.97(0.88,1.06)^b^	0.001	54.8	**0.89(0.82,0.98)** b	0.024	41.1
Hospital based	1540/1928	**0.65(0.53,0.79)**	0.111	50	0.85(0.72,1.01)	0.868	0	**0.78 (0.67,0.92)**	0.585	0	**0.74(0.63,0.87)**	0.092	53.5

a P value of Q-test for heterogeneity test.

b Random-effects model was used when a P value<0.05 for heterogeneity test; otherwise, fixed-effects model was used.

*I* ^2^: 0–25, no heterogeneity; 25–50, modest heterogeneity; 50, high heterogeneity.

For the rs2910164 polymorphism, no significant risk association was observed in the overall pooled analysis. However, cancer type-subgroup analysis revealed a decreased risk for the comparison of CC vs. GG in the subgroup of HCC (OR = 0.76, 95% CI: 0.59–0.99, *P*_h_ = 0.313), prostate cancer (OR = 0.77, 95% CI: 0.65–0.91, *P*_h_ = 0.425), cervical cancer (OR = 0.50, 95% CI: 0.37–0.68, *P*_h_ = 0.814) and esophageal cancer (OR = 0.58, 95% CI: 0.37–0.90, *P*_h_ = 0.055). Similarly, a decreased risk was observed for the comparison of GC vs. GG in the cervical cancer (OR = 0.71, 95% CI: 0.51–0.99, *P*_h_ = 0.254), CC+GC vs. GG in esophageal cancer (OR = 0.79, 95% CI: 0.65–0.96, *P*_h_ = 0.195), and CC vs. GG+GC in prostate cancer (OR = 0.65, 95% CI: 0.44–0.96, *P*_h_ = 0.699) and esophageal cancer (OR = 0.64, 95% CI: 0.41–0.98, *P*_h_ = 0.079). Subgroup analysis by ethnicity revealed a decreased risk in the Asian population (CC vs. GG: OR = 0.80, 95% CI: 0.67–0.96, *P*_h_ = 0.000; GC vs. GG: OR = 0.91, 95% CI: 0.84–0.98, *P*_h_ = 0.139; CC+GC vs. GG: OR = 0.88, 95% CI: 0.79–0.99, *P*_h_ = 0.002; CC vs. GG+GC: OR = 0.86, 95% CI: 0.76–0.98, *P*_h_ = 0.000) but not Caucasian population. A decreased risk was also observed for the comparison of CC vs. GG in both studies based population (OR = 0.87, 95% CI: 0.77–0.98, *P*_h_ = 0.000) and hospital based controls (OR = 0.65, 95% CI: 0.53–0.79, *P*_h_ = 0.000) when performed subgroup analysis by the source of controls. In contrast, an increased risk was also observed in the other cancers group for the comparison of CC+GC vs. GG (OR = 1.09, 95% CI: 1.00–1.19, Z = 2.02, *P* = 0.043, *P*_h_ = 0.222) as summarized in Table 3.

**Table 3:** Stratification analyses of genetic susceptibility of rs2910164 polymorphism to cancer risk.

Category	cases/controls	CC vs. GG			GC vs. GG			CC+GC vs. GG			CC vs. GG+GC		
		OR(95% CI)	*P* a	*I* ^2^	OR(95% CI)	*P* a	*I* ^2^	OR (95% CI)	*P* a	*I* ^2^	OR(95% CI)	*P* a	*I* ^2^
Total	13751/16838	0.88(0.75,1.03)^b^	0	68	0.98(0.90,1.06)^b^	0.005	46.4	0.94(0.86,1.02)^b^	0	58.7	0.91(0.81,1.02)^b^	0	63.9
Cancer types													
HCC	1146/1500	**0.76(0.59,0.99)**	0.313	15.9	0.92(0.70,1.21)	0.208	0	0.87(0.71,1.07)	0.169	37.9	0.88(0.74,1.05)	0.371	6.3
Gastric cancer	3125/4533	0.92(0.63,1.34)^b^	0	84.1	0.96(0.79,1.16)	0.136	45.8	0.96(0.74,1.24)	0.011	73.1	0.92(0.70,1.21)^b^	0	83.5
Breast cancer	3007/3718	1.11(0.93,1.33)	0.497	0	1.01 (0.90,1.11)	0.538	0	1.03(0.93,1.14)	0.587	0	1.06(0.92,1.23)	0.331	9.6
Prostate cancer	410/510	**0.77(0.65,0.91)**	0.425	0	0.90(0.58,1.41)	0.131	56.1	0.97(0.92,1.02)	0.062	71.4	**0.65(0.44,0.96)**	0.699	0
Cervical cancer	673/752	**0.50(0.37,0.68)**	0.814	0	**0.71(0.51,0.99)**	0.254	23.1	0.82(0.65,1.04)	0.382	0	0.65(0.72,1.11)	0.359	0
Esophageal cancer	772/779	**0.58(0.37,0.90)**	0.055	72.9	0.82(0.66,1.01)	0.406	0	**0.79(0.65,0.96)**	0.195	40.4	**0.64(0.41,0.98)**	0.079	67.6
Other cancers	4618/5046	1.06 (0.81,1.40)^b^	0.021	55.6	1.07(0.94,1.22)	0.05	48.3	1.09(1.00,1.19)	0.222	24.9	1.03(0.77,1.36)^b^	0.003	65.5
Ethnicities													
Asian	8531/10645	**0.80(0.67,0.96)** b	0	69	**0.91(0.84,0.98)**	0.139	27.1	**0.88(0.79,0.99)** ^c^	0.002	55.5	**0.86(0.76,0.98)** b	0	62.9
Caucasian	4781/5715	1.06(0.79,1.43)^b^	0.027	55.6	1.07(0.93,1.22)^b^	0.03	55	1.07(0.99,1.16)	0.243	23.4	1.03(0.74,1.44)^b^	0.005	65.9
Source of controls													
Population based	10187/11827	**0.87(0.77,0.98)** b	0	65.3	0.97(0.88,1.06)^b^	0.008	47.1	0.95(0.86,1.04)^b^	0.001	55.1	0.89(0.78,1.03)^b^	0	78.8
Hospital based	3564/5011	**0.65(0.53,0.79)** b	0	80.6	0.99(0.93,1.06)	0.089	50.5	1.00(0.80,1.25)^b^	0.005	73.2	0.95(0.74,1.21)^b^	0.001	60.3

a P value of Q-test for heterogeneity test.

b Random-effects model was used when a P value<0.05 for heterogeneity test; otherwise, fixed-effects model was used.

*I* ^2^: 0–25, no heterogeneity; 25–50, modest heterogeneity; 50, high heterogeneity.

For the rs3746444 polymorphism, there was no significant risk association observed for the overall pooled analysis of cancer risk. However, increased risks were observed for GG vs. AA (OR = 1.23, 95% CI: 1.00–1.50, Z = 2.00, *P* = 0.045, *P*_h_ = 0.118), GA vs. AA (OR = 1.19, 95% CI: 1.01–1.41, *P*_h_ = 0.001) and CC+GC vs. GG (OR = 1.14, 95% CI: 1.05–1.25, *P*_h_ = 0.003) in the Asian population rather than in the Caucasian population summarized in Table 4. For the rs2292832, there was no significant association observed in all comparisons (data not shown).

**Table 4:** Stratification analyses of genetic susceptibility of rs3746444 polymorphism to cancer risk.

Category	cases/controls	GG vs. AA			GA vs. AA			GG+GA vs. AA			GG vs. GA+AA		
		OR(95% CI)	*P* a	*I* ^2^	OR(95% CI)	*P* a	*I* ^2^	OR (95% CI)	*P* a	*I* ^2^	OR(95% CI)	*P* a	*I* ^2^
Total	7025/8427	1.11(0.95,1.29)	0.127	32	1.12(0.97,1.29)^b^	0	69.8	1.12(0.98,1.28)^b^	0	68.2	1.06(0.91,1.23)	0.07	39.1
Cancer types													
HCC	445/784	1.25(0.36,4.34)^b^	0.023	73.6	1.00(0.76,1.31)^b^	0.074	61.6	1.12(0.63,1.99)^b^	0.009	78.8	1.51(0.87,2.62)	0.06	64
Breast cancer	2588/3260	1.26(0.70,2.26)^b^	0.036	77.2	1.07 (0.95,1.20)	0.163	48.6	1.08(0.97,1.20)	0.056	72.7	1.11(0.87,1.42)	0.05	74
Other cancers	3992/4383	1.06(0.85,1.28)	0.795	0	1.17(0.94,1.46) b	0	78.4	1.14(0.95,1.36)^b^	0.001	71.2	0.98(0.80,1.20)	0.31	15.4
Ethnicities													
Asian	4337/5130	**1.23(1.00,1.50)**	0.118	35	**1.19(1.01,1.41)** b	0.001	65	**1.14(1.05,1.25)** b	0.003	62.1	1.08(0.81,1.44)^b^	0.04	47.2
Caucasian	2688/3297	0.97(0.76,1.22)	0.97	0	0.93(0.83,1.04)	0.053	73.2	0.92(0.76,1.11)	0.083	66.8	0.99(0.78,1.24)	0.74	0

a P value of Q-test for heterogeneity test.

b Random-effects model was used when a P value<0.05 for heterogeneity test; otherwise, fixed-effects model was used.

*I* ^2^: 0–25, no heterogeneity; 25–50, modest heterogeneity; 50 high heterogeneity.

### Test of heterogeneity

There was significant heterogeneity across the studies of the rs11614913, rs2910164, rs3746444, and thus the source of heterogeneity was further explored by the heterozygote comparison. For the rs11614913, cancer type (χ2 = 23.68, df = 5, *P* = 0.000) and source of control (χ2 = 5.63, df = 1, *P* = 0.018) were the source of the heterogeneity. For rs2910164 polymorphism, cancer type (χ2 = 27.65, df = 6, *P* = 0.000) and ethnicity (χ2 = 15.52, df = 3, *P* = 0.000) contributed substantially to the heterogeneity. For the rs3746444 polymorphism, ethnicity (χ2 = 8.38, df = 1, *P* = 0.004) contributed substantially to heterogeneity.

Sensitivity analysis revealed that the four independent studies [14], [15], [16], [17] were the main cause of heterogeneity for the rs11614913. Heterogeneity was decreased when these studies were removed (TT+CT vs. CC: *P*_h_ = 0.061, *I* ^2^ = 33.49%). Similarly, heterogeneity of the rs2910164 (CC+GC vs. GG: *P*_h_ = 0.060, *I* ^2^ = 33.5%) and rs3746444 (GG+GA vs. AA: *P*_h_ = 0.092, *I* ^2^ = 39.8%) were decreased when the four [18], [19], [20], [21] and the three [16], [22], [23] independent studies removed, respectively.

### Publication bias

**Figure 1:**
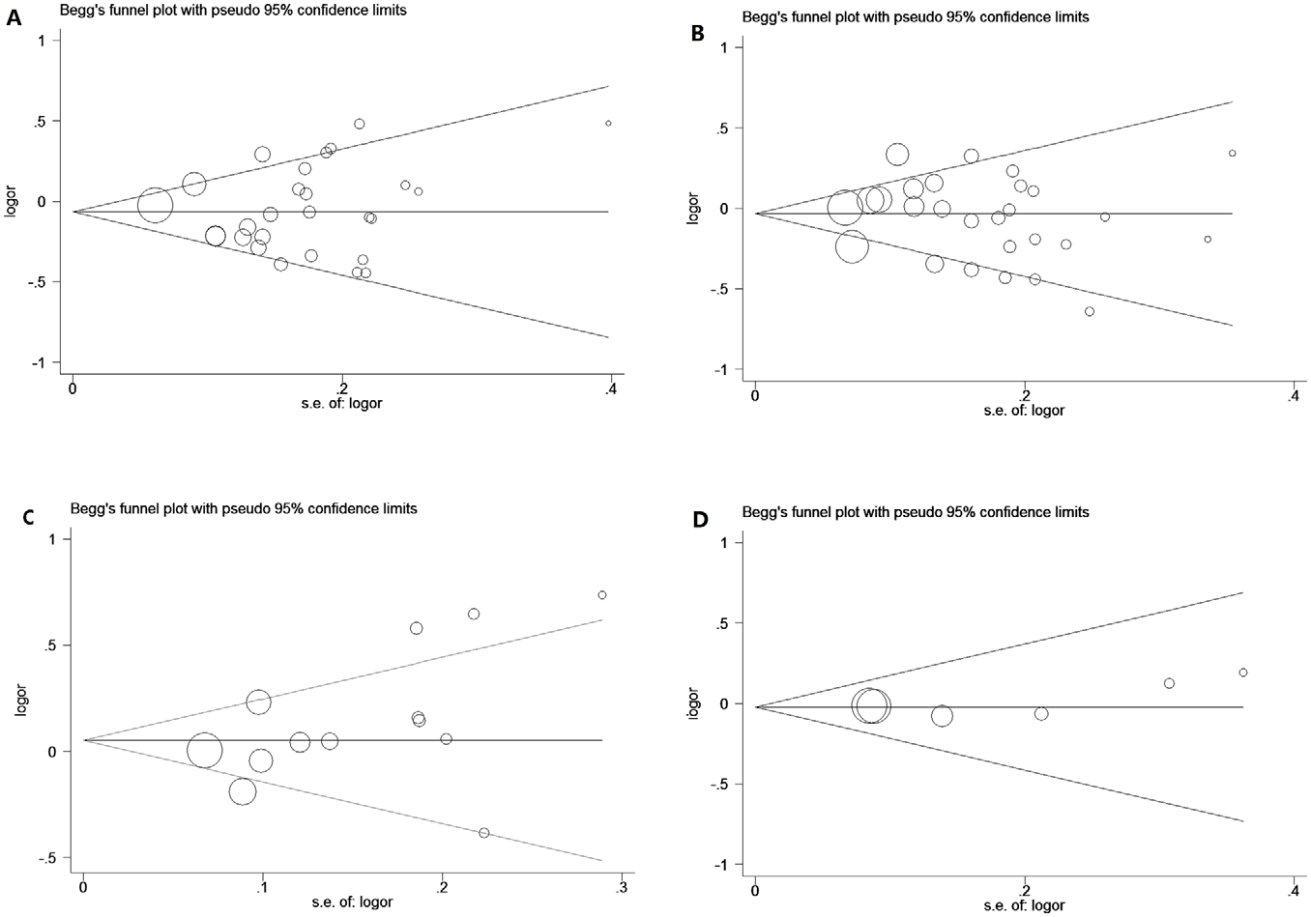
Begg's funnel plot for publication bias test. Each circle denotes an independent study for the indicated association. Log[OR], natural logarithm of OR. Horizontal line stands for mean effect size. A: rs11614913, B: rs2910164, C: rs37464444, D: rs2292832.

Begg's funnel plot and Egger's test were performed to assess the publication bias of the currently available literature. The shape of the funnel plots did not reveal any evidence of obvious asymmetry in all comparison models. Then, the Egger's test was used to provide statistical evidence for funnel plot symmetry. The results also did not show any evidence of publication bias (rs11614913: *t* = 0.25, *P* =  0.806, rs2910164: *t* = −0.70, *P* = 0.489, rs37464444: *t* = 1.88, *P* = 0.087, and rs2292832: *t* = 1.14, *P* = 0.318 for dominant model. Figure 1).

## Discussion

In this meta-analysis, an association between the four common SNPs in microRNAs (rs11614913, rs2910164, rs3746444, and rs2292832) and cancer risk was evaluated by the pooled results from 40 published studies. The results demonstrated that the rs11614913TT genotype was associated with a decreased risk for developing cancer, in particular for colorectal cancer and lung cancer, or in the Asian population, and that the rs2910164C allele was associated with a decreased risk for developing esophageal cancer, cervical cancer, prostate cancer and HCC, in particular in the Asian population. Contrary to the above, the rs3746444G allele was observed as a risk factor for cancer in the Asian population; however, the rs2292832 polymorphism was not associated with cancer risk.

The rs11614913 polymorphism present in the miR-196a2 has significantly greater impact on miR-196a expression and is associated with various carcinogenesis [24], [25], [26]. Although there were studies reporting no direct association between rs11614913 and the expression of miR-196a [9], [13], previous, meta-analysis studies have suggested an association between rs11614913 and risk of cancers [7], [27], [28], [29], This updated meta-analysis further support the rs11614913 TT genotype was associated with a decreased risk for cancer. In addition, significant associations were observed in the Asian population but not in the Caucasian population, suggesting a possible ethnic difference in the genetic background and the environment, which was the similar to that reported by Chu et al [28] and Wang et al [27]. In contrast to the published pooled results, this updated pooled results revealed that the rs116114913 TT could be a protective factor against colorectal cancer and lung cancer. However, no significant association was observed in breast cancer, suggesting that carcinogenic mechanisms may differ in the tumor sites and hsa-miR-196a2 genetic variants.. The risk of different cancer types should be confirmed by more studies.

For the rs2910164, no significant association was observed in overall pooled results, as supported by the report by Xu et al [7]. In contrast to the published results, this study revealed the different association between rs2910164 polymorphism and cancer risk among ethnicity and the cancer types. The rs2910164 CC genotype was associated with decreased risk for esophageal cancer, cervical cancer, prostate cancer, and HCC in the Asian population, suggesting a difference in genetic background and the environment, and pathogenesis of different tumor sites. The rs2910164 in the miR-146aG>C gene is located in the stem region opposite to the mature miR-146 sequence and results in a change from G∶U pair to C∶U mismatch in the stem structure of miR-146a precursor. It has been reported that the G-allelic miR-146a precursor could increase the production of mature miR-146a and affecting target mRNA binding [18], [19].

The rs3746444 polymorphism present in the miR-499 would target to SOX6 and Rod1 genes important roles for the etiology of cancers [30], [31]. The pooled results from 13 studies revealed that rs3746444G allele was associated with an increased risk for developing cancer in the Asian population. To our knowledge, this is the first meta-analysisabout the association of rs3746444 of cancer from 11 Asian population studies and two Caucasian population studies. More studies should be accumulated to confirm the results. The rs2292832 polymorphism has also been evaluated by six enrolled studies, with no significant associations were found from all pooled results. Thus far, few epidemiologic studies have investigated the association of rs2292832 polymorphism and cancer risk.

The heterogeneity were observed across the studies for the polymorphisms of rs11614913, rs2910164, rs3746444, the source of the heterogeneity were mainly from the cancer type, such as glioma, gallbladder, bladder, and papillary thyroid carcinoma and cervical cancer, suggesting polymorphisms in miRNAs may play different roles according the cancer type. Furthermore, different risk of polymorphisms in miRNAs was also the source of the heterogeneity, significant associations were observed in the most studies for Asian populations. The studies based on different source of control were also the source of the heterogeneity of studies.

Although meta-analysis is robust, our study still has some limitations. First, our meta-analysis did not evaluate any potential gene-gene interaction and gene-environment interaction due to lack of relevant published data. Second, although all eligible studies were summarized, the relatively small sample size of studies may lead to reduced statistical power when stratified according to the tumor type, ethnicity or infection status. Last, relatively large heterogeneity was observed across the all studies involved.

In summary, this meta-analysis suggested that the rs11614913TT genotype was associated with a decreased cancer risk, especially for colorectal cancer and lung cancer, that the rs2910164C allele was a protective factor for esophageal cancer, cervical cancer, prostate cancer and HCC, and that the rs11614913, rs2910164, and rs3746444 SNPs were risk factors for cancer in the Asian population.

## Supporting Information

Figure S1 **Process of study selection of case–control studies.**
(DOC)

## References

[1] Bartel DP (2004) MicroRNAs: genomics, biogenesis, mechanism, and function. Cell 116: 281–297. 14744438 10.1016/s0092-8674(04)00045-5

[2] Berezikov E, Guryev V, van de Belt J, Wienholds E, Plasterk RH, et al (2005) Phylogenetic shadowing and computational identification of human microRNA genes. Cell 120: 21–24. 15652478 10.1016/j.cell.2004.12.031

[3] Calin GA, Croce CM (2006) MicroRNA signatures in human cancers. Nat Rev Cancer 6: 857–866. 17060945 10.1038/nrc1997

[4] Cho WC (2010) MicroRNAs: potential biomarkers for cancer diagnosis, prognosis and targets for therapy. Int J Biochem Cell Biol 42: 1273–1281. 20026422 10.1016/j.biocel.2009.12.014

[5] Cho WC (2010) Recent progress in genetic variants associated with cancer and their implications in diagnostics development. Expert Rev Mol Diagn 10: 699–703. 20843192 10.1586/erm.10.64

[6] Ryan BM, Robles AI, Harris CC (2010) Genetic variation in microRNA networks: the implications for cancer research. Nat Rev Cancer 10: 389–402. 20495573 10.1038/nrc2867 PMC2950312

[7] Xu W, Xu J, Liu S, Chen B, Wang X, et al (2011) Effects of common polymorphisms rs11614913 in miR-196a2 and rs2910164 in miR-146a on cancer susceptibility: a meta-analysis. PLoS One 6: e20471. 21637771 10.1371/journal.pone.0020471 PMC3102728

[8] Horikawa Y, Wood CG, Yang H, Zhao H, Ye Y, et al (2008) Single nucleotide polymorphisms of microRNA machinery genes modify the risk of renal cell carcinoma. Clin Cancer Res 14: 7956–7962. 19047128 10.1158/1078-0432.CCR-08-1199 PMC2650498

[9] Christensen BC, Avissar-Whiting M, Ouellet LG, Butler RA, Nelson HH, et al (2010) Mature microRNA sequence polymorphism in MIR196A2 is associated with risk and prognosis of head and neck cancer. Clin Cancer Res 16: 3713–3720. 20501619 10.1158/1078-0432.CCR-10-0657 PMC2914465

[10] Handoll HH (2006) Systematic reviews on rehabilitation interventions. Arch Phys Med Rehabil 87: 875. 16731227 10.1016/j.apmr.2006.04.006

[11] Midgette AS, Wong JB, Beshansky JR, Porath A, Fleming C, et al (1994) Cost-effectiveness of streptokinase for acute myocardial infarction: A combined meta-analysis and decision analysis of the effects of infarct location and of likelihood of infarction. Med Decis Making 14: 108–117. 8028463 10.1177/0272989X9401400203

[12] Egger M, Davey Smith G, Schneider M, Minder C (1997) Bias in meta-analysis detected by a simple, graphical test. BMJ 315: 629–634. 9310563 10.1136/bmj.315.7109.629 PMC2127453

[13] Hoffman AE, Zheng T, Yi C, Leaderer D, Weidhaas J, et al (2009) microRNA miR-196a-2 and breast cancer: a genetic and epigenetic association study and functional analysis. Cancer Res 69: 5970–5977. 19567675 10.1158/0008-5472.CAN-09-0236 PMC2716085

[14] Dou T, Wu Q, Chen X, Ribas J, Ni X, et al (2010) A polymorphism of microRNA196a genome region was associated with decreased risk of glioma in Chinese population. J Cancer Res Clin Oncol 136: 1853–1859. 20229273 10.1007/s00432-010-0844-5 PMC11827793

[15] Srivastava K, Srivastava A, Mittal B (2010) Common genetic variants in pre-microRNAs and risk of gallbladder cancer in North Indian population. J Hum Genet 55: 495–499. 20520619 10.1038/jhg.2010.54

[16] George GP, Gangwar R, Mandal RK, Sankhwar SN, Mittal RD (2011) Genetic variation in microRNA genes and prostate cancer risk in North Indian population. Mol Biol Rep 38: 1609–1615. 20842445 10.1007/s11033-010-0270-4

[17] Mittal RD, Gangwar R, George GP, Mittal T, Kapoor R (2011) Investigative role of pre-microRNAs in bladder cancer patients: a case-control study in North India. DNA Cell Biol 30: 401–406. 21345130 10.1089/dna.2010.1159

[18] Xu T, Zhu Y, Wei QK, Yuan Y, Zhou F, et al (2008) A functional polymorphism in the miR-146a gene is associated with the risk for hepatocellular carcinoma. Carcinogenesis 29: 2126–2131. 18711148 10.1093/carcin/bgn195

[19] Jazdzewski K, Murray EL, Franssila K, Jarzab B, Schoenberg DR, et al (2008) Common SNP in pre-miR-146a decreases mature miR expression and predisposes to papillary thyroid carcinoma. Proc Natl Acad Sci U S A 105: 7269–7274. 18474871 10.1073/pnas.0802682105 PMC2438239

[20] Okubo M, Tahara T, Shibata T, Yamashita H, Nakamura M, et al (2010) Association between common genetic variants in pre-microRNAs and gastric cancer risk in Japanese population. Helicobacter 15: 524–531. 21073609 10.1111/j.1523-5378.2010.00806.x

[21] Zhou F, Zhu H, Luo D, Wang M, Dong X, et al (2012) A Functional Polymorphism in Pre-miR-146a Is Associated with Susceptibility to Gastric Cancer in a Chinese Population. DNA Cell Biol 31: 1290–1295. 22455393 10.1089/dna.2011.1596

[22] Zhou B, Wang K, Wang Y, Xi M, Zhang Z, et al (2011) Common genetic polymorphisms in pre-microRNAs and risk of cervical squamous cell carcinoma. Mol Carcinog 50: 499–505. 21319225 10.1002/mc.20740

[23] Xiang Y, Fan S, Cao J, Huang S, Zhang LP (2012) Association of the microRNA-499 variants with susceptibility to hepatocellular carcinoma in a Chinese population. Mol Biol Rep 39: 7019–7023. 22311030 10.1007/s11033-012-1532-0

[24] Hu Z, Chen J, Tian T, Zhou X, Gu H, et al (2008) Genetic variants of miRNA sequences and non-small cell lung cancer survival. J Clin Invest 118: 2600–2608. 18521189 10.1172/JCI34934 PMC2402113

[25] Zhan JF, Chen LH, Chen ZX, Yuan YW, Xie GZ, et al (2011) A functional variant in microRNA-196a2 is associated with susceptibility of colorectal cancer in a Chinese population. Arch Med Res 42: 144–148. 21565628 10.1016/j.arcmed.2011.04.001

[26] Li XD, Li ZG, Song XX, Liu CF (2010) A variant in microRNA-196a2 is associated with susceptibility to hepatocellular carcinoma in Chinese patients with cirrhosis. Pathology 42: 669–673. 21080878 10.3109/00313025.2010.522175

[27] Wang F, Ma YL, Zhang P, Yang JJ, Chen HQ, et al (2012) A genetic variant in microRNA-196a2 is associated with increased cancer risk: a meta-analysis. Mol Biol Rep 39: 269–275. 21625865 10.1007/s11033-011-0735-0

[28] Chu H, Wang M, Shi D, Ma L, Zhang Z, et al (2011) Hsa-miR-196a2 Rs11614913 polymorphism contributes to cancer susceptibility: evidence from 15 case-control studies. PLoS One 6: e18108. 21483822 10.1371/journal.pone.0018108 PMC3069063

[29] Qiu LX, Wang Y, Xia ZG, Xi B, Mao C, et al (2011) miR-196a2 C allele is a low-penetrant risk factor for cancer development. Cytokine 56: 589–592. 21907588 10.1016/j.cyto.2011.08.019

[30] Qi P, Dou TH, Geng L, Zhou FG, Gu X, et al (2010) Association of a variant in MIR 196A2 with susceptibility to hepatocellular carcinoma in male Chinese patients with chronic hepatitis B virus infection. Hum Immunol 71: 621–626. 20188135 10.1016/j.humimm.2010.02.017

[31] Tano K, Mizuno R, Okada T, Rakwal R, Shibato J, et al (2010) MALAT-1 enhances cell motility of lung adenocarcinoma cells by influencing the expression of motility-related genes. FEBS Lett 584: 4575–4580. 20937273 10.1016/j.febslet.2010.10.008

