# Supplementary material for: The Association between Four Genetic Variants in MicroRNAs (rs11614913, rs2910164, rs3746444, rs2292832) and Cancer Risk: Evidence from Published Studies
Source: PLoS One. 2012 Nov 14;7(11):e49032. doi: 10.1371/journal.pone.0049032 (PMC3498348; doi:10.1371/journal.pone.0049032)
Supplement: Figure S1 — Process of study selection of case–control studies. (DOC) [file pone.0049032.s001.doc]

Figure S1 Process of study selection of case–control studies

Additional records identified through reference list searching
(n = 21)

Records after duplicates removed
(n = 298)

Records screened
(n =298)

Records excluded
(n =221)

Full-text articles assessed for eligibility
(n = 77)

Full-text articles excluded, for reviews, no data(n=37)

Studies included in qualitative synthesis
(n = 40)

Studies included in quantitative synthesis

Records identified through database searching
(n =293)
